# The protective effect of hydrogen sulfide on systemic sclerosis associated skin and lung fibrosis in mice model

**DOI:** 10.1186/s40064-016-2774-4

**Published:** 2016-07-15

**Authors:** Zhi Wang, Xiaoya Yin, Luyan Gao, Sheng Feng, Kai Song, Lingyun Li, Ying Lu, Huaying Shen

**Affiliations:** Department of Nephrology, Second Affiliated Hospital of Soochow University, 1055 Sanxiang Road, Jinchang, Suzhou, 215000 Jiangsu Province China

**Keywords:** Systemic sclerosis, Fibrosis, Transforming growth factor-β1, Hydrogen sulfide

## Abstract

**Backgroud:**

Systemic sclerosis (SSc) caused fibrosis can be fatal and it still lack of effective treatment. Hydrogen sulfide (H_2_S) appears to be an attractive therapeutic candidates. This study aimed to investigate the protective effect of H_2_S on SSc-associated skin and lung fibrosis.

**Methods:**

We developed a model of SSc by subcutaneous injecting BLM to female C3H mice. The mice received daily subcutaneous injections of NaHS (56 and 112 μg/kg), an H_2_S donor. On days 7, 28, and 42, the mice were killed and blood samples were collected to measure the plasma H_2_S concentration, the skin and lung tissues was harvested for microscopic examination, immunohistochemistry and quantify biological parameters (hydroxyproline content, RT-qPCR and Western blot).

**Results:**

In model group, the dermis of skin tissues at different time points gradually thickened, collagen deposition increased. The lung tissues presented pathological changes such as obvious inflammatory cell infiltration, increased collagen deposition and the plasma H_2_S concentrations points significantly decreased. Administration of NaHS markedly decreased the biomarkers of fibrosis such as α-smooth muscle actin, collagen-I, collagen-III, fibronectin, transforming growth factor-β1, Smad2/3 phosphorylation and inflammation including the marker protein of monocyte/macrophage and monocyte chemoattractant protein-1 in the lung. Compared to the low dose group, the expression in the high dose group have decreased trend, but the difference was not significant.

**Conclusion:**

We demonstrate the beneficial effects of H_2_S on SSc-associated skin and lung fibrosis. H_2_S may be a potential therapy against this intractable disease.

## Background

Systemic sclerosis (SSc) is a severe connective tissue disease of unknown etiology. SSc is characterized by multivisceral fibrosis resulting from inflammation, vascular injury and excessive collagen deposition. Skin sclerosis can cause discomfort and organ involvements—such as pulmonary fibrosis—can be fatal. However, the mechanisms of fibrosis in SSc have not been fully elucidated and effective drugs are still scarce (Walker et al. 2012). Immunosuppressants, used to treat SSc, can also cause important adverse effects, such as hepatic and renal functional lesion, bone marrow depressions and infecting etc. Therefore, new therapy options need to be found.

Hydrogen sulfide (H_2_S) is a newly recognized endogenous gasotransmitter analogic to nitric oxide and carbon monoxide (Wang 2012). As a new emerging gaseous signalling molecule, for a long time, its study was limited to its toxicity. In the 1990s, it was discovered that endogenous H_2_S participated in anti-inflammation, anti-oxidation, and the regulation of cell proliferation and apoptosis (Gao et al. 2012; Moody and Calvert 2011; Vandiver and Snyder 2012). Past studies have confirmed that exogenous H_2_S attenuates aortic-coarctation-induced cardiac hypertrophy and fibrosis (Huang et al. 2012), inhibit renal interstitial fibrosis caused by obstructive nephropathy (Song et al. 2014), relieve peritoneal mesothelial cell injury caused by high-glucose peritoneal dialysis fluids (Lu et al. 2015) and carbon tetrachloride-induced cirrhosis (Tan et al. 2011). The potential mechanisms of H_2_S anti-fibrotic may be account for inhibiting the activation and migration of inflammatory cells likewise myofibroblasts, subsequently decrease the production of pro-inflammatory cytokines and extracellular matrix accumulation (Li et al. 2008; Tan et al. 2011). Therefore, we hypothesized that H_2_S may against organ fibrosis caused by immune reaction. Importantly, the present of H_2_S in a variety of mammalian tissues and organs support it may be without overt adverse effect.

We conduct this study to investigate the effect of H_2_S on SSc-associated skin and lung fibrosis in mice model established by subcutaneous injections with BLM, and also examined the underlying anti-fibrotic mechanisms.

## Methods

### Establishment of animal models

After adaptive feeding for 1 week, 75 clean-grade female C3H mice with a body weight of 18–22 g (SLAC, Shanghai) were randomly divided into 5 groups: (1) control group: local subcutaneous injection of PBS 0.1 ml + intraperitoneal injection of PBS 1 ml; (2) model group: local subcutaneous injection of 1 mg/ml bleomycin (BLM, Nippon Kayaku Co. Ltd.) 0.1 ml + intraperitoneal injection of PBS 1 ml; (3) low dose treatment group: local subcutaneous injection of 1 mg/ml BLM 0.1 ml + intraperitoneal injection of 56 μg/kg/d NaHS (Sigma, USA); (4) high dose treatment group: local subcutaneous injection of 1 mg/ml BLM 0.1 ml + intraperitoneal injection of 112 μg/kg/d NaHS; (5) H_2_S group: local subcutaneous injection of PBS 0.1 ml + intraperitoneal injection of 112 μg/kg/d NaHS. BLM (0.1 ml) dissolved in PBS at a concentration of 1 mg/ml, was subcutaneously each day for 4 consecutive weeks. Intraperitoneal injection of 56 μg/kg/d or 112 μg/kg/d NaHS began at the same time with BLM injection and lasted for 6 weeks. Five mice in each group were randomly sacrificed on days 7, 28, and 42. Serum samples of mice were collected to measure the H_2_S concentrations. Skin and lung tissues of mice were collected for pathological staining usinghaematoxylin-eosin (HE) stain and Masson stain to observe the degrees of inflammation and fibrosis in lung tissues. The alkaline hydrolysis method was performed to determine the concentrations of hydroxyproline (HYP) in lung tissues (Jiangcheng Bioengineering Institute, Nanjing). PCR and western blot were performed to detect the expression of fibrosis indicators such as α-smooth muscle actin (α-SMA), collagen-I (Col-I), collagen-III (Col-III), fibronectin (FN), transforming growth factor-β1 (TGF-β1) and Smad2/3 phosphorylation (p-Smad2/3). Immunohistochemistry was performed to detect the expression of inflammatory cytokines including the marker protein (ED-1) of monocyte/macrophage and monocyte chemoattractant protein-1 (MCP-1). The α-SMA, FN and p-Smad2/3 antibodies antibodies were purchased from Santa Cruz; all other antibodies were purchased from Abcam.

### Histopathological staining

Skin tissues and left lung tissues from the injection locations in the neck and back of mice were fixed in 4 % paraformaldehyde, embedded in paraffin, and cut into 5 μm sections. HE and Masson stains were performed to observe the changes of gross morphology, inflammation, and collagen deposition.

### Measurement of the plasma H_2_S levels

The NaHS standard was used for a twofold serial dilution to obtain concentrations from 1.56 to 100 μM. The DNA-Az (H_2_S probe) was dissolved in anhydrous ethanol to obtain the final concentration of 2.2 mM. One black 96-well plate was obtained, and 10 μl of 2.2 mM DNS-Az stock solution was then added toeach well followed by 100 μl fresh plasma or the NaHS standard immediately to obtaina DNS-Az concentration of 200 μM. The absorbance value of each well was measured in a microplate reader. The excitation wavelength was 360 nm, and the emission wavelength was 528 nm. The NaHS results were used to plot a standard curve. The absorbance values of plasma wells were compared with the standard curve to obtain the plasma H_2_S levels.

### Measurement of HYP concentration

Lung tissues of mice at a wet weight of 30–100 mg were collected. The HYP levels in lung tissues were detected using alkaline hydrolysis colorimetry. Lung tissues were hydrolysed using an alkaline solution. Chloramine T, perchloric acid, and 4-(dimethylamino) benzaldehyde were sequentially added. After mixing thoroughly, samples were incubated in a 60 °C water bath for 15 min. The samples were cooled down and centrifuged, and the supernatant was collected. The absorbance values at 550 nm wavelength and 1 cm optic path were measured. Calibration was performed using a blank tube and a standard tube. The results arepresented as μg/mg.

### RT-PCR

Total RNA from right lung tissues of mice was extracted using the TRIzol method. The concentration of the total RNA was measured using a spectrophotometer. The synthesis of cDNA was performed according to the proceduresof the reverse transcription reagent kit. The primer sequences of β-actin, α-SMA, Col-I, and Col-III were synthesized by Sangon Biotech (Shanghai). Primer sequences are shown in Table 1.

**Table 1:** Primers for polymerase chain reaction

cDNA	Primers	Reaction condition
β-actin	5′-GTGCTATGTTGCTCTAGACTTCG-3′	94 °C, 30 s; 55 °C, 30 s; 72 °C, 1 min (30)
	5′-ATGCCACAGGATTCCATACC-3′	
α-SMA	5′-GGGAGTAATGGTTGGAATGG-3′	94 °C, 30 s; 55 °C, 30 s; 72 °C, 1 min (30)
	5′-GGTGATGATGCCGTGTTCTA-3′	
Col-1	5′-TGACTGGAAGAGCGGAGAGT-3′	94 °C, 30 s; 52 °C, 30 s; 72 °C, 1 min (30)
	5′-CGGCTGAGTAGGGAACACAC-3′	
Col-III	5′-AGGTTCTCCTGGTGCTGCT-3′	94 °C, 30 s; 55 °C, 30 s; 72 °C, 1 min (30)
	5′-ATGCCCACTTGTTCCATCTT-3′	

*α*-*SMA* α-smooth muscle actin, *Col*-*1* collagen-I, *Col*-*III* collagen-III

### Western blot

The frozen right lung tissues of mice were added to the tissue lysis solution. After homogenization and centrifugation, the supernatant was collected. Protein concentrations were determined using the BCA method, and the protein samples were stored in a −80 °C freezer for future usage. A 10 % polyacrylamide gel was prepared for electrophoresis. After the samples were transferred to a membrane and blocked, the primary antibody was added (1:1000 dilution) and incubated at 4 °C overnight. The secondary antibody was then added (1:5000 dilution) and incubated at room temperature for 1 h. The results were developed, and fibrosis indicators such as α-SMA, FN, TGF-β1 and p-Smad2/3 were detected.

### Immunohistochemistry

The streptavidin–biotin peroxidase complex (ABC) method was used for immunohistochemistry. The dilution of the primary antibodies was 1:100 for ED1and 1:100 for MCP-1. Five fields were randomly selected under a microscope (400×). After the positive regions were localized, images were taken using a colour camera. The results were input into a colour image analysis system. After the positive regions were precisely segmented, the quantities of ED1 and MCP-1 positive cells were measured, and the mean values were calculated.

### Statistical analysis

Measurement data are presented as mean ± SD. Comparison among multiple groups was performed using one-way analysis of variance. A threshold α = 0.05 was used as the detection level. Data were analysed using the SPSS17.0 software.

## Results

### Pathological changes in skin tissues

**Fig. 1:**
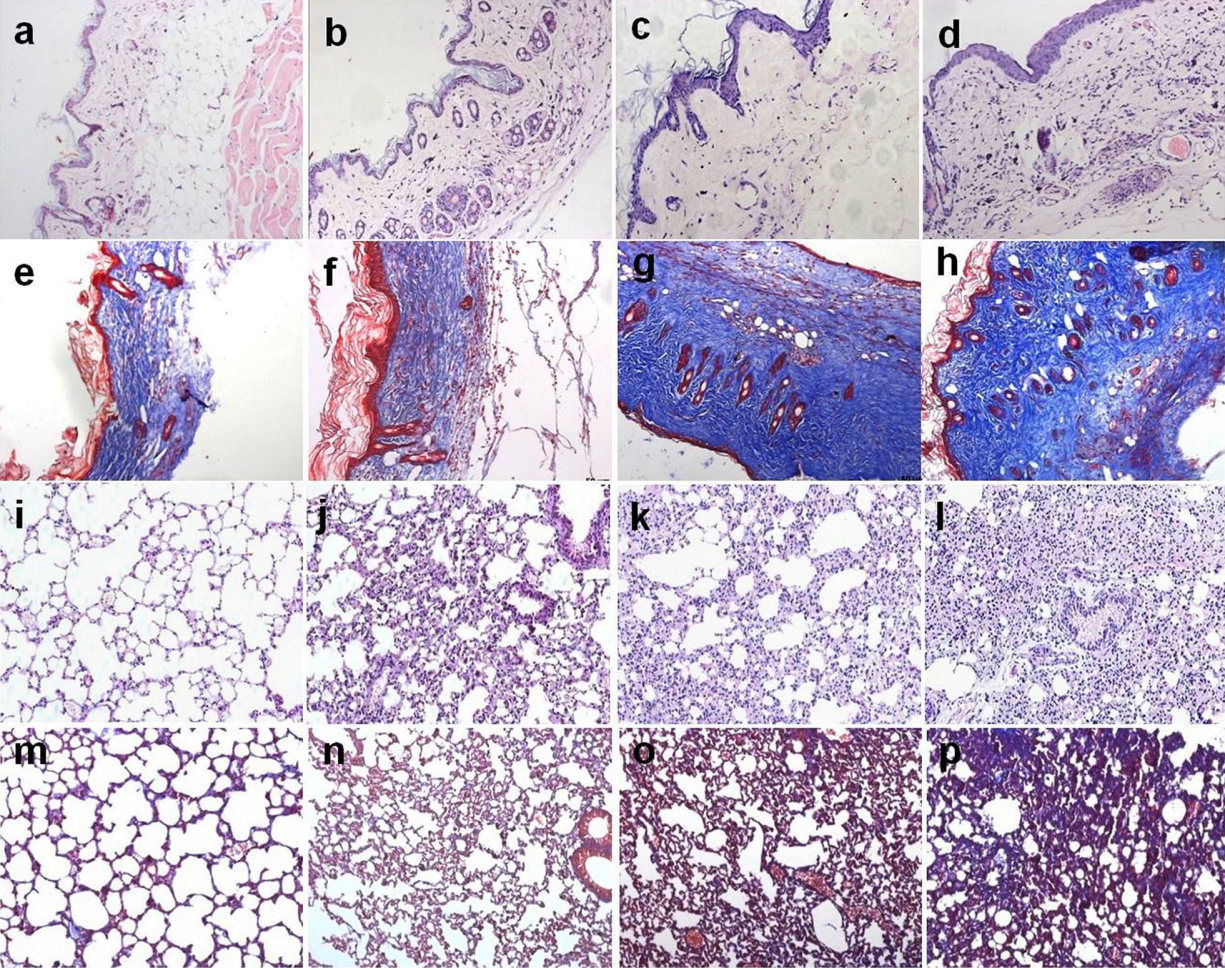
HE stain and Masson stain results of skin and lung tissues in mice (×200). **a**–**d**, **i**–**l** HE stain; **e**–**h**, **m**–**p** Masson stain, **a**–**h** Skin tissues, **i**–**p** Lung tissues. **a**, **e**; **i**, **m** In the control group, the skin tissues had proper thickness and clear structure; the lung tissues indicated that the alveoli of mice were transparent and bright, the structure was intact. **b**, **f**; **j**, **n** In the 7-day Model group, the skin tissues showed little thickening and the inflammatory cell infiltration was the most pronounced; the inflammatory cell infiltration was more evident. **c**, **g**; **k**, **o** In the 28-day Model group, skin samples from the systemic sclerosis mice showed significant thickening; the lung tissues increased collagen deposition, and thickening of alveolar septa. **d**, **h**; **l**, **p**. In the 42-day Model group, the collagen deposition and skin fibrosis were pronounced; alveolar collapse, collagen deposition, and pulmonary parenchymal fibrosis evident

**Fig. 2:**
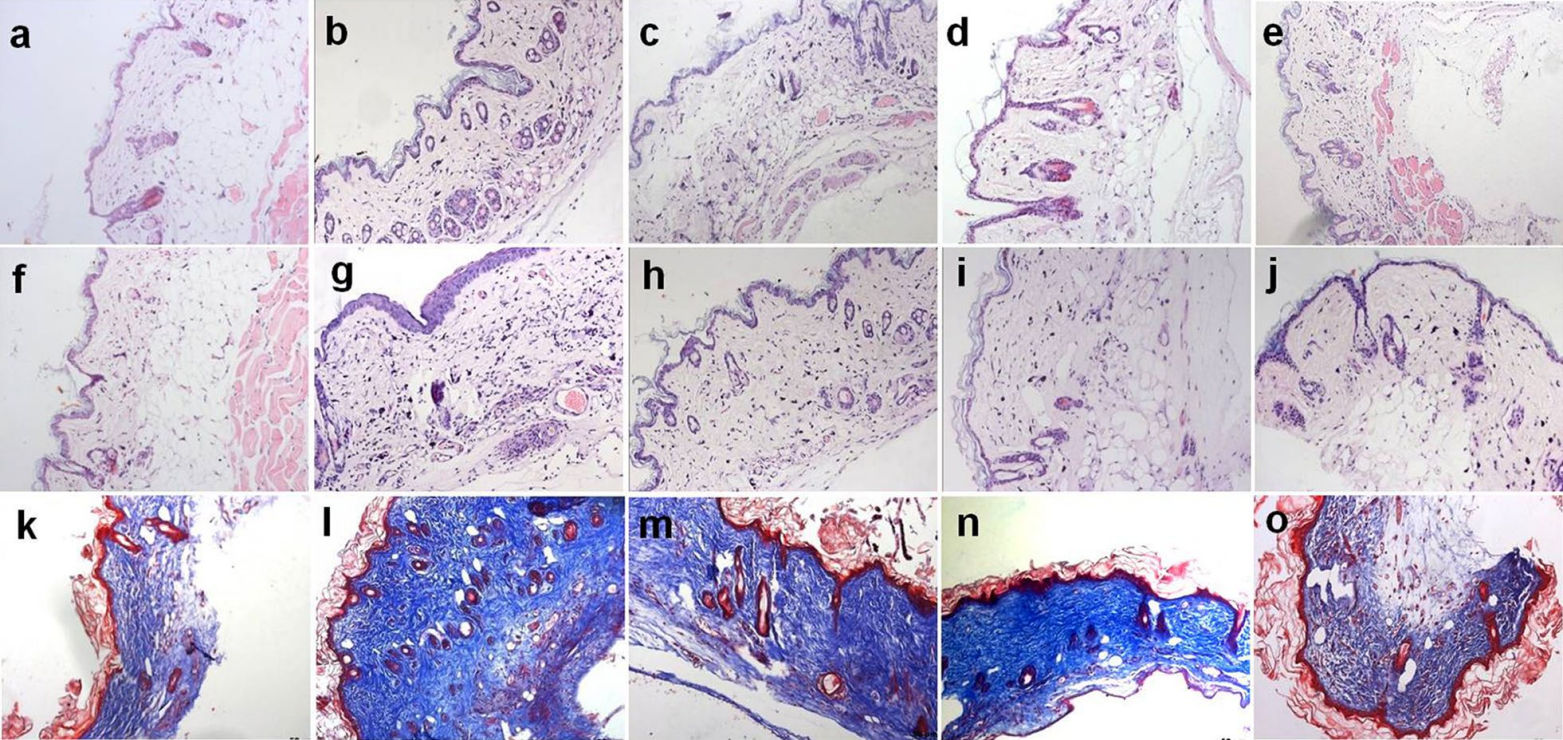
H_2_S improves pathological changes of skin tissues in BLM-induced mice (×200). **a**–**j** HE stain; **k**–**o** Masson stain. **a**–**e** 7-day group; **f**–**o** 42-day group; **a**, **f**, **k** In the control group, the skin tissues had proper thickness and clear structure; **b**, **g**, **l** In the 7-day and 42-day model group, skin tissues points exhibited significant thickening of the dermis and disordered structure with increasing model establishment time; **c**, **h**, **m** and **d**, **i**, **n** In the low and high dose treatment group, the skin tissues exhibited a clear structure, the dermis became thinner, the lipid layer thickened, inflammatory cell filtration and collagen deposition significantly decreased compared to the model group; **e**, **j**, **o** Repeated injections of NaHS had little effect compared with the control group

HE stain and Masson stain results of skin tissues indicated that the dermis of mice in the control group had proper thickness, clear structure, and a large amount of underlying adipose tissue (Fig. 1a, e). Skin tissues of mice in the model group at different time points exhibited significant thickening of the dermis and disordered structure with increasing model establishment timethere was inflammatory cell filtration and collagen deposition (Fig. 1b–d, f–h). Of these features, the inflammatory cell infiltration was the most pronounced in the 7-day group (Fig. 1b), and the collagen deposition and skin fibrosis were the more pronounced in the 28- and 42-day group (Fig. 1g, h). After NaHS intervention, the skin tissues of mice in the treatment group exhibited a clear structure, the dermis became thinner, the lipid layer thickened, inflammatory cell filtration and collagen deposition significantly decreased compared to the model group on 7-day (Fig. 2c, d) and on 42 day (Fig. 2h, i; m, n). Compared to that in the low dose treatment group, the improvement in the high dose group was even more clearly detectable (Fig. 2e, j, o).

### Pathological changes in lung tissues

**Fig. 3:**
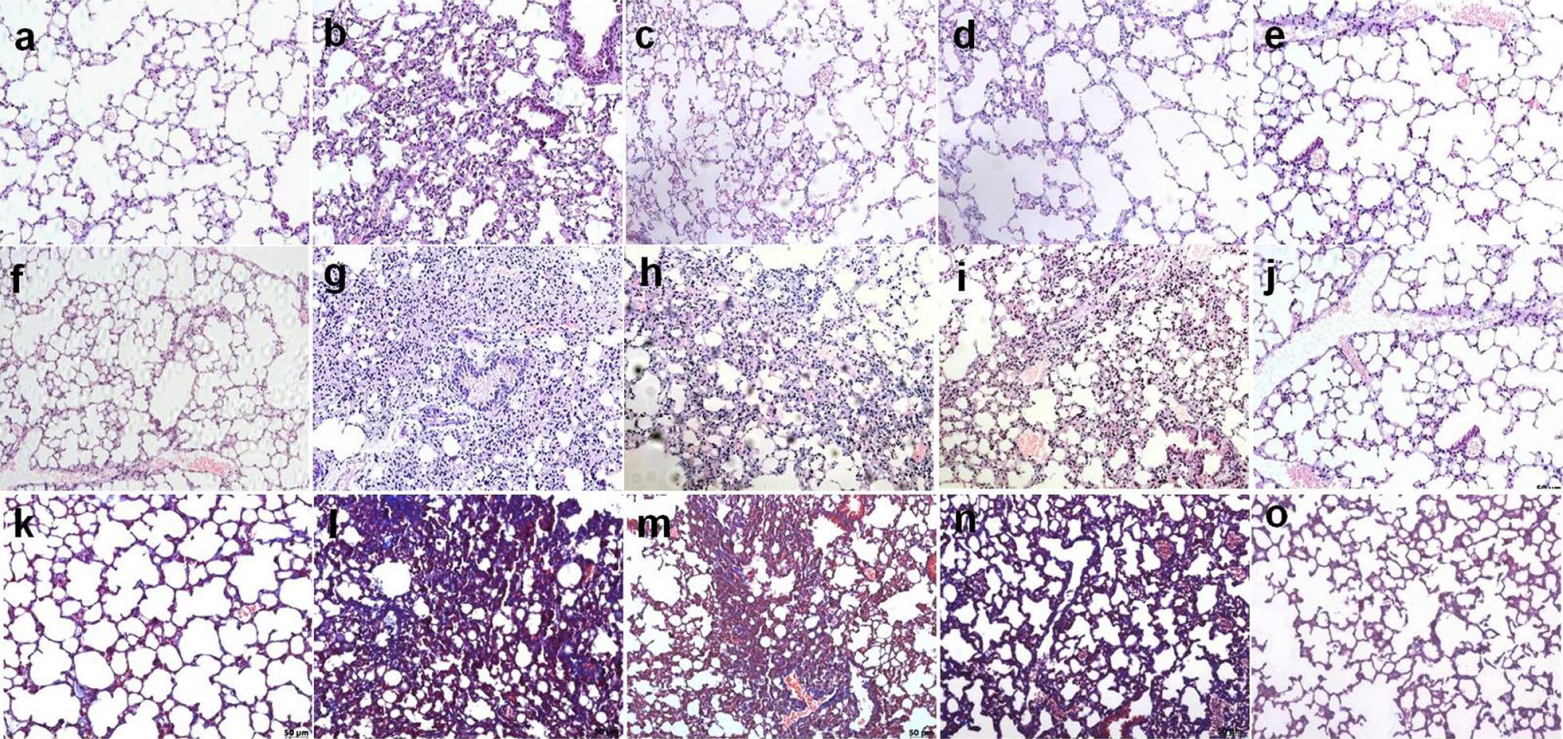
H_2_S improves pathological changes of lung tissues in BLM-induced mice (×200). **a**–**j** HE stain; **k**–**o** Masson stain. **a**–**e** 7-day group; **f**–**o** 42-day group; **a**, **f**, **k** In the control group, the lung tissues were transparent and bright; **b**, **g**, **l** In the 7-day and 42-day model group, points all exhibited obvious inflammatory cell infiltration, disordered structure, increased collagen deposition, and thickening of alveolar septa; **c**, **h**, **m** and **d**, **i**, **n** In the low and high dose treatment group, the above targets significantly improved. **e**, **j**, **o** Repeated injections of NaHS had no significant effect on pathological changes of lung tissues

HE stain and Masson stain results of lung tissues indicated that the alveoli of mice in the control group were transparent and bright, the structure was intact (Fig. 1i, m). Compared to the control group, the model group at different time points all exhibited obvious inflammatory cell infiltration, alveolar collapse, disordered structure, increased collagen deposition, and thickening of alveolar septa (Fig. 1j–l; m, o, p). Thereinto, the inflammatory cell infiltration was more evident in the 7-day group (Fig. 1j); whereas the alveolar collapse, patchy fusion, collagen deposition, and pulmonary parenchymal fibrosis were the more evident in the 28- and 42-day group (Fig. 1o, p). After NaHS intervention, lung tissue pathological changes in mice from the treatment group were significantly relieved, inflammatory cell filtration and collagen deposition decreased, alveolar structure improved, the alveoli were more transparent and bright, and the degree of inflammation and fibrosis decreased on 7-day (Fig. 3c, d) and on 42 day (Fig. 3h, i; m, n).

### Changes in the plasma H_2_S concentrations

**Fig. 4:**
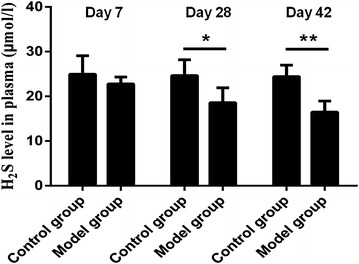
Changes in the plasma H_2_S concentrations. Compared to the control group, the plasma H_2_S concentrations for mice in the model group at different time points all decreased. Results represent mean ± SD of three independent experiments. ^*^ *P* < 0.05 versus untreated group; ^**^ *P* < 0.01 versus untreated group

Compared to the control group, the plasma H_2_S concentrations for mice in the model group at different time points all decreased. The mean plasma H_2_S concentration for mice in the 7-day control group was 24.96 ± 4.12 μM. The concentration in the 7-day model group was 22.79 ± 1.54 μM; the concentration decreased compared to that in the control group, but the difference was not statistically significant. The concentrations in the 28- and the 42-day control groups were 24.66 ± 3.52 and 24.41 ± 2.57 μM, The concentrations in the 28-day model group and the 42-day model groups were 18.59 ± 3.32 and 16.49 ± 2.46 μM, respectively compared to that in the control group, the differences were each statistically significant (Fig. 4).

### H_2_S reduces the expression level of HYP in the lung tissues of mice induced by BLM on the 42th day

**Fig. 5:**
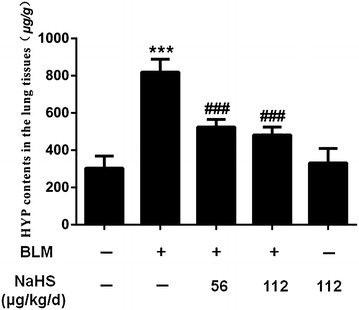
H_2_S reduces the expression level of HYP in the lung tissues of mice induced by BLM on the 42th day. Compared to the control group, the model group points significantly increased; After NaHS intervention, the HYP concentration in the treatment group significantly decreased. The decreasing trend in the high dose treatment group was more evident than that in the low dose treatment group. Data are presented as the mean ± SD. ^***^ *P* < 0.001 versus control group. ^*###*^ *P* < 0.001 versus modle group

Compared to that in the control group, the HYP levels in the lung tissues of mice in the model group at different time points all significantly increased. For example in the 42-day group, after NaHS intervention, the HYP concentration in the treatment group significantly decreased. The decreasing trend in the high dose treatment group was more evident than that in the low dose treatment group, but the difference was not statistically significant (Fig. 5).

### H_2_S inhibits the expression of α-SMA, Col-I, Col-III and FN in lung tissues on the 42th day

**Fig. 6:**
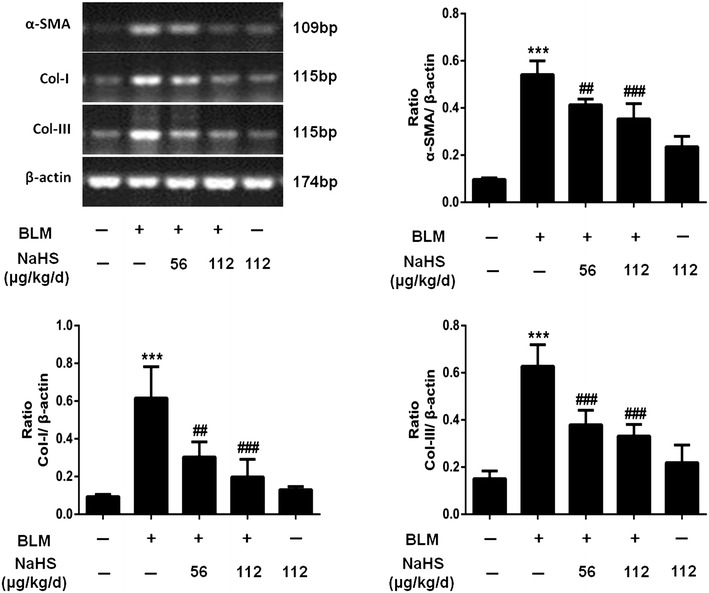
H_2_S inhibits the expression of α-SMA, Col-I and Col-III in lung tissues on the 42th day. α-SMA, Col-I and Col-III mRNA were semi-quantified. Results represent mean ± SD of three independent experiments. ^***^ *P* < 0.001 versus untreated group; ^###^ *P* < 0.001 versus modle group; ^##^ *P* < 0.01 versus modle group

Compared to that in the control group, the mRNA expression levels of α-SMA, Col-I, and Col-III in the lung tissues of mice in the model group at different time points were significantly upregulated. For example in the 42-day group, after NaHS intervention, the expression levels of the above indicators for mice in the treatment group were significantly downregulated, and the differences were statistically significant. The trend of downregulation in the high dose treatment group was more evident than that in the low dose treatment group, but the difference was not statistically significant (Fig. 6).

**Fig. 7:**
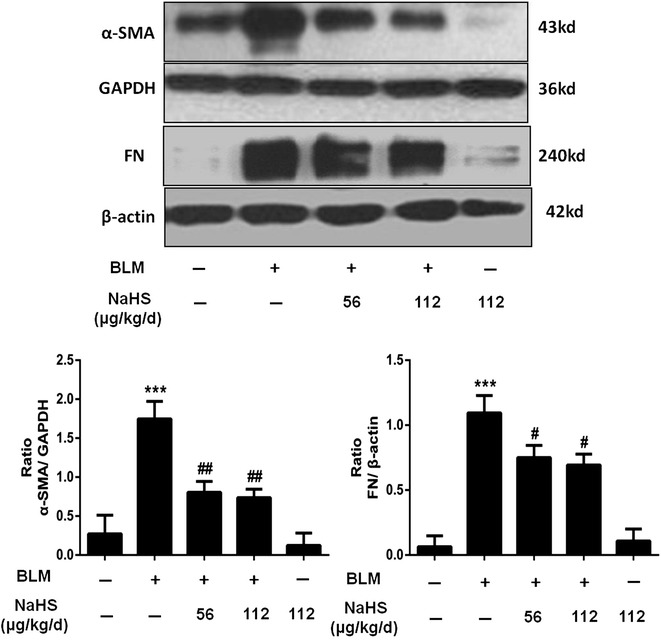
H_2_S reduces the expression of α-SMA and FN in lung tissues on the 42th day. After NaHS intervention, the expression levels of α-SMA and FN in the lung tissues were significantly downregulated, the trend of downregulation of the expression levels of the above proteins in the high dose treatment group was more evident, but the difference was not statistically significant. Results represent mean ± SD of three independent experiments. ^***^ *P* < 0.001 versus untreated group; ^#^ *P* < 0.05 versus modle group; ^##^ *P* < 0.01 versus modle group

Compared to that in the control group, the protein expression levels of α-SMA and FN in the lung tissues of mice in the model group were significantly upregulated, and in the 42-day group after NaHS intervention, the expression levels of the above indicators for mice in the treatment group were significantly downregulated, and the differences were statistically significant. Compared to that in the low dose treatment group, the trend of downregulation of the expression levels of the above proteins in the high dose treatment group was more evident, but the difference was not statistically significant (Fig. 7).

### H_2_S reduces the number of ED-1-positive cells and MCP-1-positive cells in lung tissues on the 7th day

**Fig. 8:**
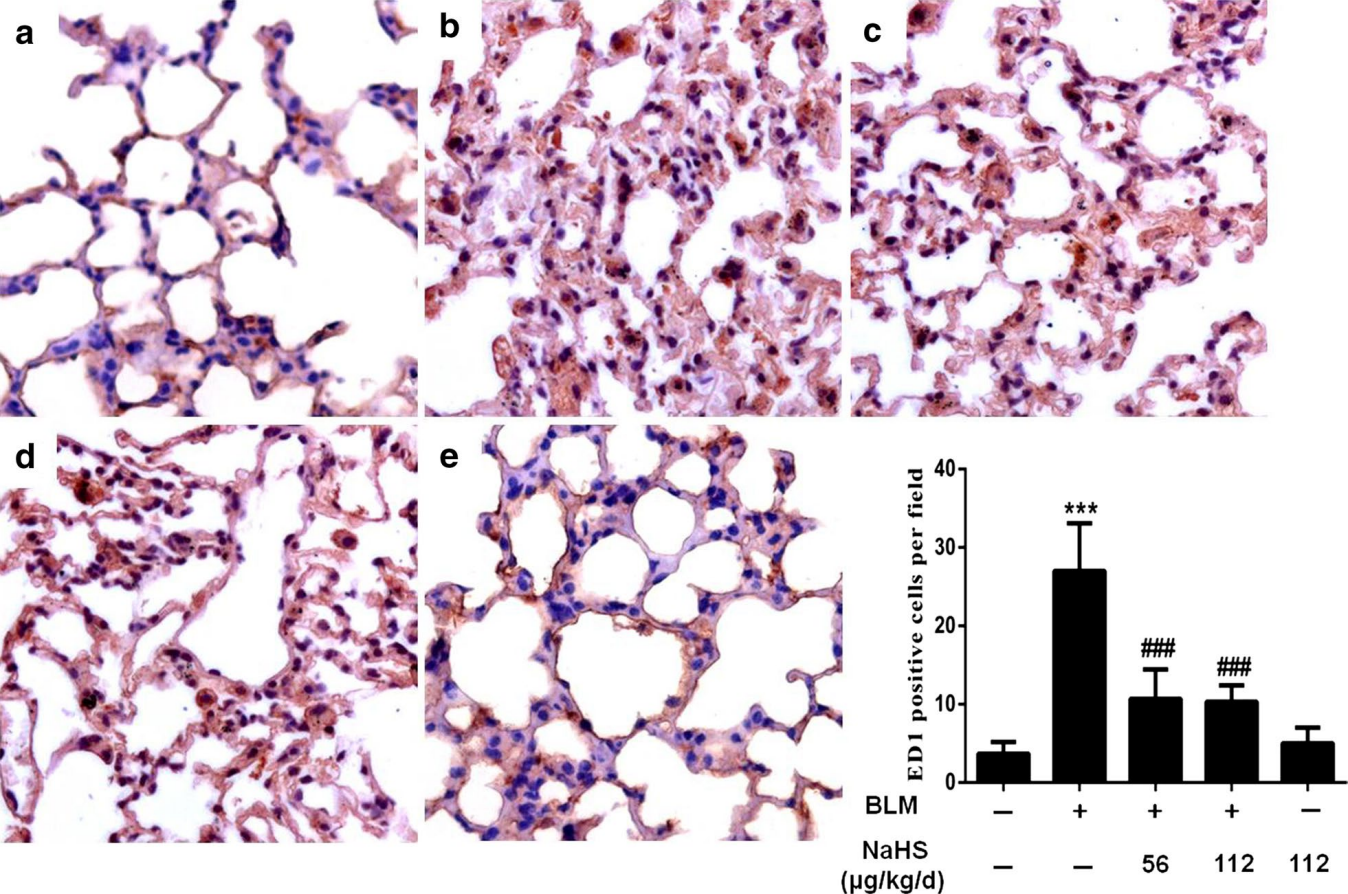
H_2_S reduces the number of ED-1-positive cells in lung tissues on the 7th day. **a**–**e** Immunohistochemistry staining of ED-1. **a** In the control group, ED-1-positive cells in the interstitial tissue were rare; **b** In the model group, the number of ED-1-positive cells were significantly increased; **c**, **d** In the treatment group, comparison with the model group, the RA contained significantly fewer ED-1-positive cells in the lung tissues; **e** In the NaHS group, ED-1-positive cells were equivalent to that of control mice. Data are presented as the mean ± SD. ^***^ *P* < 0.001 versus control group. ^*###*^ *P* < 0.001 versus modle group

**Fig. 9:**
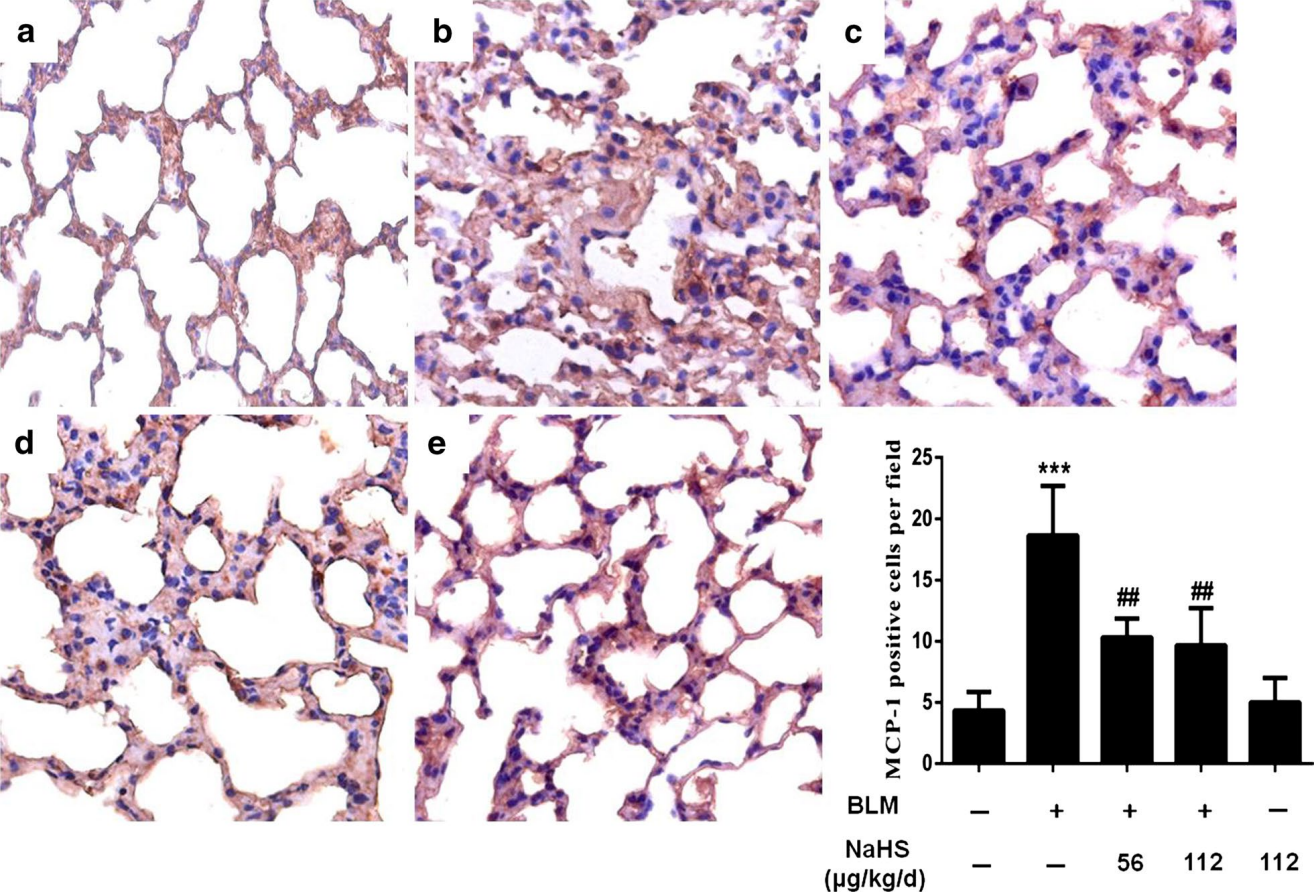
H_2_S reduces the number of MCP-1-positive cells in lung tissues on the 7th day. **a**–**e** Immunohistochemistry staining of MCP-1. **a** In the control group, few MCP-1-positive cells were present in the lung tissues. **b** In the model group, note the strong expression of MCP-1-positive cells in the markedly thickened submesothelial compact zone; **c**, **d** In the treatment group, comparison with the model group, the racontained significantly fewer MCP-1-positive cells in the lung tissues; **e** In the NaHS group, MCP-1-positive cells were similar to that of control mice. Data are presented as the mean ± SD. ^***^ *P* < 0.001 versus control group. ^###^ *P* < 0.001 versus modle group

Alveolitis was the most clearly observed in the 7-day model group. Therefore, the 7-day group was used for routine ED1 and MCP-1 immunohistochemistry to investigate the effects of H_2_S on inflammatory cytokines. The results of immunohistochemistry indicated that compared to the control group, the 7-day model group exhibited significant inflammatory cell infiltration and an increased number of ED1 and MCP-1 positive expression cells (Figs. 8b, 9b). After NaHS intervention, the expression levels of the above positive cells were significant downregulated (Figs. 8c, d, 9c, d). However, the difference between the low dose treatment group and the high dose treatment group was not statistically significant.

### H_2_S inhibits the expression of TGF-β1 and p-Smad2/3 in lung tissues on the 28th day and the 42th day

**Fig. 10:**
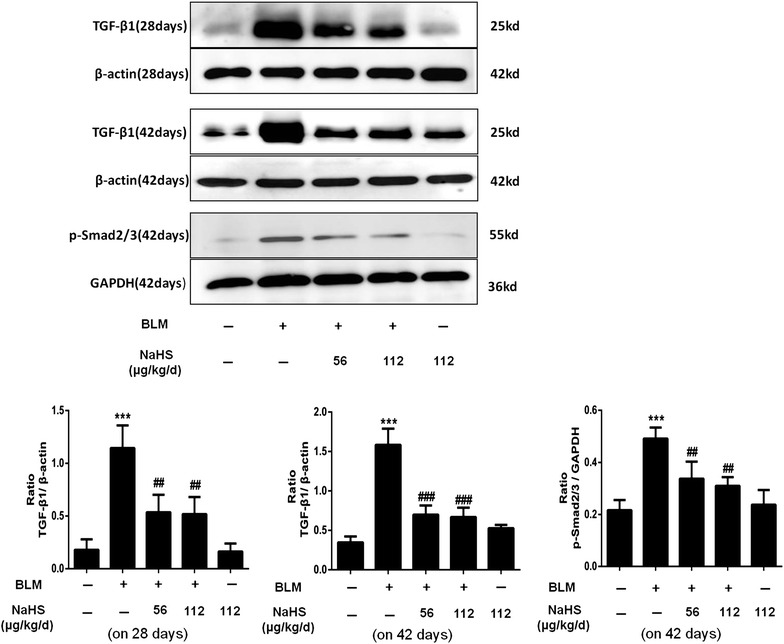
H_2_S inhibits the expression of TGF-β1 and p-Smad2/3 in lung tissues. After NaHS intervention, the expression levels of TGF-β1and p-Smad2/3 in the lung tissues were significantly downregulated, the trend of downregulation of the expression levels of the above proteins in the high dose treatment group was more evident, but the difference was not statistically significant. Results represent mean ± SD of three independent experiments. ^***^ *P* < 0.001 versus untreated group; ^###^ *P* < 0.001 versus modle group; ^##^ *P* < 0.01 versus modle group

Compared to the control group, the protein expression levels of TGF-β1 in lung tissues of mice in the model group at different time points were significantly upregulated, particularly the 28- and 42-day groups. After NaHS intervention, the protein expression level of TGF-β1 of mice in the treatment group was significantly downregulated, and the difference was statistically significant; however, the difference between the low dose treatment group and the high dose treatment group was not statistically significant. Meanwhile, compared to the control group, the protein expression levels of p-Smad2/3 in the 42 days model group lung tissues were significantly upregulated. After NaHS administration, the protein expression level of p-Smad2/3 in the treatment group was significantly downregulated, and the difference was statistically significant (Fig. 10).

## Discussion

The present study demonstrated that inflammatory cell infiltration in skin and lung tissues increases and the synthesis of fibrogenic cytokines increases in a BLM-induced SSc mice model. Previous studies have suggested that exogenous H_2_S exerted a protective effect of pulmonary fibrosis by attenuating lipid peroxidation injury (Fang et al. 2009). And this study confirmed that exogenous H_2_S could relieve SSc through an anti-inflammation and anti-fibrosis mechanism, which provided a novel therapeutic approach to SSc.

In the present study, the mice model of SSc was established by subcutaneous injection of BLM. Consistent with previous studies (Yamamoto and Nishioka 2002; Yamamoto et al. 1999), we could observe the destruction of the structure of skin and lung tissues, such as disordered structure, increased inflammatory cell infiltration, collagen deposition, significant skin and pulmonary fibrosis in mice from the model group at different time points. Furthermore, studies confirmed that the skin and lung tissues of SSc mice exhibited increased inflammatory cell filtration and upregulation of TGF-β1 expression at the early stage (Bienkowski and Gotkin 1995; Bonniaud et al. 2005). This study confirmed that BLM could induce inflammatory cell filtration and increase of the expression of fibrogenic factors in skin and lung tissues, these findings were consistent with previous studies indicated that the SSc mice model induced by BLM in this study was successfully established.

The relation of H_2_S and SSc associated fibrosis is not well defined. The results of this study suggested that the plasma H_2_S levels are decreased in the model group after subcutaneous injection of BLM. Which was consistent with the conclusion of previous studies showing that “organ fibrosis might be associated with H_2_S deficiency” (El-Seweidy et al. 2011; Tan et al. 2011). Therefore, we investigate the role of H_2_S on SSc-associated skin and lung fibrosis.

Our study indicate that H_2_S exhibited an anti-inflammatory property by reducing macrophage recruitment in lung tissues. The inflammatory cytokines such as ED-1 and MCP-1 were downregulated by NaHS in the 7-day group. Substantial data have suggested that H_2_S is protective against inflammation in multiple fibrosis-related diseases (Babaei-Karamshahlou et al. 2012; Huang et al. 2012; Lu et al. 2015; Song et al. 2014; Whiteman and Winyard 2011). Our team previous study indicated that administration at a relatively low dose (56 μg/kg/d) exogenous H_2_S could inhibit renal interstitial mononuclear cell infiltration and TNF-α expression in a unilateral ureteral obstructive (UUO) model, suggesting that H_2_S could exert its anti-inflammation function through the inhibition of the activation of macrophages (Song et al. 2014). Therefore, we speculated that the anti-pulmonary fibrosis function of H_2_S was associated with its anti-inflammation function. Inflammation plays an important role at the initial stage of systemic sclerosis, and inflammatory cell infiltration is the upstream event and one of the significant pathological features of organ fibrosis. The above study indicated that H_2_S inhibits the progression of skin and lung fibrosis through its anti-inflammation function.

In addition to the factor of inflammatory reaction, cytokines particular TGF-β1 also play an important role in the occurrence and development of fibrosis diseases (Flanders and Burmester 2003; Pittet et al. 2001). Substantial data have demonstrated that TGF-β1 promotes the transdifferentiation of fibroblasts into myofibroblasts (Scotton and Chambers 2007). Our results showed that NaHS reduced the expression level of HYP and suppressed mRNA and protein expressions of a-SMA, FN, Col-1and Col-3 induced by BLM in the lung tissues. Importantly, NaHS also attenuated the protein expressions of TGF-β1 at 28 and 42 days. These data suggested that H_2_S inhibited the fibroblast differentiation and extracellular matrix production partially by blocking the expression of TGF-β1. It is well known that TGF-β1 to be working main through its signal protein p-Smad2/3. Our study showed that after NaHS administration, the protein expression level of p-Smad2/3 in the treatment group was significantly downregulated on day 42. These results consistent with the previous study (Bei et al. 2013). This finding suggests that the anti-fibrotic effect of H_2_S may be connected with its inhibition of TGF-β/Smads pathway.

The above study confirmed that exogenous H_2_S had a protective function on SSc-related organ fibrosis. And importantly the present of H_2_S in a variety of mammalian tissues and organs support it may be without overt adverse effect. However, currently acquirable H_2_S donors like NaHS used in present study are not suitable for therapy because they release H_2_S uncontrolled. Tries are being made to obtain more appropriate H_2_S precursors for therapeutic aim. GYY4137 and SG1002 are two administrated H_2_S-releasing compounds that have been proven to be beneficial in various diseases such as hepatocellular carcinoma, diabetes, and chronic heart failure (Kondo et al. 2013; Lu et al. 2014; Wei et al. 2014). Such compounds may become useful for treatment of SSc-related organ fibrosis.

## Conclusions

In conclusions, this study confirmed that H_2_S could improve SSc-related organ fibrosis. The mechanism was achieved through the inhibition of the inflammatory reaction and the reduction of TGF-β1 expression, thus reducing the accumulation of extracellular matrix. This study provides new insight into the treatment of SSc-related organ fibrosis in the clinic. A detailed understanding of the molecular mechanism and the related cell signalling pathways still awaits further study.
